# Editorial: Natural compounds and novel sources of antimicrobial agents for food preservation and biofilm control, volume II

**DOI:** 10.3389/fmicb.2024.1412881

**Published:** 2024-05-10

**Authors:** Lizziane Kretli Winkelströter, Eugenia Bezirtzoglou, Fabricio Luiz Tulini

**Affiliations:** ^1^Health Sciences Faculty, UNOESTE (University of Western São Paulo), São Paulo, Brazil; ^2^Laboratory of Hygiene and Environmental Protection, Department of Medicine, Democritus University of Thrace, Alexandroupolis, Greece; ^3^Center for Biological and Health Sciences (CCBS), Federal University of Western Bahia, Barreiras, Brazil

**Keywords:** biofilms, foodborne pathogens, food safety, biocontrol, food additives

Natural compounds and novel sources of antimicrobial agents are gaining increasing attention as effective means of controlling biofilm and food preservation. With the rise of antibiotic resistance and consumer demand for natural and sustainable food products, there is a growing interest in exploring alternative antimicrobial strategies derived from natural sources (Mishra et al., 2020). Researchers are investigating several natural compounds, such as plant extracts, essential oils, and antimicrobial peptides, in order to evaluate their potential to inhibit the growth of foodborne pathogens and disrupt biofilm formation in food processing environments. These compounds offer promising alternatives to synthetic antimicrobials, with the added benefit of being biodegradable and environmentally friendly (Mishra et al., 2020; Rather et al., 2021).

One significant advantage of natural compounds is their multifaceted mode of action against microbial pathogens. Unlike conventional antimicrobials, which often target specific cellular components, natural compounds can exhibit broad-spectrum antimicrobial activity by affecting multiple physiological pathways in bacteria (Rather et al., 2021; Liu et al., 2023). Furthermore, the exploration of novel sources of antimicrobial agents from untapped natural reservoirs holds a promising potential for discovering potent compounds with unique properties. By harnessing the biochemical diversity of these natural sources, scientists aim to identify novel antimicrobial compounds with enhanced potency, stability, and specificity on targeting foodborne pathogens and biofilms. Through ongoing research and innovation in natural product discovery and development, the field of antimicrobial food preservation continues to evolve, offering sustainable solutions for ensuring food safety and security in a changing global landscape (Rather et al., 2021; Rossi et al., 2022).

Researchers have conducted numerous studies to enhance comprehension on microbial biofilms and foodborne pathogens, and also to devise strategies for preventing food contamination (Mashraqi; El-Sherbiny et al.; Park et al.; Liang et al.; Yang et al.; Poscente et al.). This Research Topic outlines the formation of microbial biofilms, discusses challenges posed by food safety, outlines existing natural compounds and novel sources of antimicrobial agents for eliminating or managing harmful foodborne pathogens and microbial biofilms. In summary, ten articles were received and, after rigorous peer review process, six of them were accepted for journal publications. They focused mainly in nanocompounds, natural compounds and its combination with beneficial microorganism.

A group of manuscripts evaluated the applicability of nanocompounds. Mashraqi aimed to evaluate Dill (*Anethum graveolens* L.) extract (DE) loaded with chitosan nanoparticles (ChNPs) for controlling food-borne bacteria. The author reported that the anti-biofilm activity was 79.26 and 86.15% against *B. cereus* using DE and DELChNPs, respectively. This investigation highlighted the vital DE phytoconstituents, particularly DELChNPs, which possess important therapeutic effects against food-borne microorganisms and could be utilized as a safe alternative to synthetic drugs. Moreover, El-Sherbiny et al. tested chitosan nanoparticles (NCT), mucilage of cress seed (GCm; *Lepidium sativum*), and GCm-mediated selenium nanoparticles (GCm/SeNPs) on the production of novel bioactive natural nanocomposites (NCs) with remarkable bactericidal activity (against *Salmonella typhimurium* and *Staphylococcus aureus*). They observed that these NCs formulations could overcome the biocidal potentialities of standard biocides, as well as they could cause severe destructions/deformations in challenged *S. typhimurium* within 9 h.

The second round of manuscripts addressed the use of natural compounds, such as vitamin A1 and Proanthocyanidins (PCs). Park et al. investigated if the metabolites of vitamin A1 might diminish *S. aureus* biofilm formation and toxin production. Among the three retinoic acids that were examined, 13-cis-retinoic acid at 10 μg/ml significantly decreased *S. aureus* biofilm formation without affecting its planktonic cell growth (MIC > 400 μg/ml) and it inhibited biofilm formation by *Staphylococcus epidermidis* (MIC > 400 μg/ml). Otherwise, the inhibitory effect was lower on biofilm formation by an uropathogenic *Escherichia coli* strain, a *Vibrio* strain, or a fungal *Candida* strain. These findings suggest that metabolites of vitamin A1, particularly 13-cis-retinoic acid, might be useful on suppressing biofilm formation and virulence characteristics of *S. aureus*. In another approach, Liang et al. explored Proanthocyanidins (PCs) extracted from ume as a novel natural food preservative by the analysis of the bacteriostatic ability of UPPP and the freshness preservation effect on blueberry. The results showed that UPPP had a high inhibitory effect on *Staphylococcus aureus* (MIC of 1.563 mg/ml) and *Escherichia coli* (MIC of 3.125 mg/ml). Findings revealed that, in comparison to 0.02% potassium sorbate, blueberries treated with a high concentration of UPPP in a dipping treatment displayed superior quality maintenance after 7 days of storage at 4°C. Therefore, UPPP holds great potential as an innovative natural food preservative, effectively enhancing food safety, quality, and extending shelf life.

A combined efficacy of carvacrol and salicylic acid (SA) was also evaluated in association with *Lactobacillus plantarum* DSM 20174 and *Bacillus* sp. 1–23, respectively. Yang et al. demonstrated that *Bacillus* sp. 1–23, alone or combined with SA, could help white *Hypsizygus marmoreus* from the *Trichoderma* spp. BBP-6 infection. They reported that this strategy effectively maintain nutrients, restore and stabilize the antioxidant system, and reduce the production of malondialdehyde, superoxide anion and hydrogen peroxide. Thus, such treatments could be considered as potential methods to alleviate damage from diseases and extend the shelf life of white *H. marmoreus*. Also, Poscente et al. aimed to investigate the *in vitro* combined efficacy of carvacrol with a pre-formed biofilm monolayer of the probiotic *L. plantarum* DSM 20174. The results showed that *L. plantarum* pre-formed biofilm monolayer enhanced the antimicrobial effect of carvacrol, resulting on a bactericidal action, while the treatment alone induced the viable but not culturable (VBNC) cell state only. Therefore, the incorporation of carvacrol with *L. plantarum* pre-formed biofilm represents a promising alternative for an antimicrobial functionalized ready-to-eat packaging.

In conclusion, the exploration of new methods for controlling foodborne bacteria and biofilms presents a promising avenue for enhancing food safety and security. Natural compounds and novel antimicrobial sources offer sustainable alternatives to the traditional synthetic agents, with their multifaceted modes of action and potential to overcome microbial resistance. Thus, it is believed in a future where safe, sustainable, and nutritious food is accessible to all, mitigating the risks posed by foodborne pathogens and biofilms.

## Author contributions

LW: Writing—review & editing, Writing—original draft. EB: Writing—review & editing, Writing—original draft. FT: Writing—review & editing, Writing—original draft.
